# CXCR7 Targeting and Its Major Disease Relevance

**DOI:** 10.3389/fphar.2018.00641

**Published:** 2018-06-21

**Authors:** Chuan Wang, Weilin Chen, Jianzhong Shen

**Affiliations:** ^1^Department of Drug Discovery and Development, Harrison School of Pharmacy, Auburn University, Auburn, AL, United States; ^2^Department of Immunology, Shenzhen University School of Medicine, Shenzhen, China

**Keywords:** CXCR7, ACKR3, SDF-1, CXCL12, chemokine receptor, biased signaling

## Abstract

Chemokine receptors are the target of small peptide chemokines. They play various important roles in physiological and pathological processes. CXCR7, later renamed ACKR3, is a non-classical seven transmembrane-spanning receptor whose function as a signaling or non-signaling scavenger/decoy receptor is currently under debate. Even for cell signaling mechanisms, there has been inconsistency on whether CXCR7 couples to G-proteins or β-arrestins. Several reasons may contribute to this uncertainty or controversy. In one hand, it has been neglected that CXCR7 has more than five natural ligands and unfortunately, most of the prior research only studied SDF-1 (CXCL12) and/or I-TAC (CXCL11); on the other hand, there are mounting evidence supporting ligand and tissue bias for receptor signaling, but limited such information is available for CXCR7. In this review we focus on summarizing the endogenous and exogenous ligands of CXCR7, the main diseases related to CXCR7 and the biased signaling events happening on CXCR7. These three aspects of CXCR7 pharmacologic properties may explain why the contradicting opinions of whether CXCR7 is a signaling or non-signaling receptor exist. Further, potential new direction and perspective for the study of CXCR7 biology and pharmacology are highlighted.

## Introduction

Chemokines, with molecular weights in the range of 8 to 12 kDa, consist of about 50 small peptides in this superfamily (Rollins, 1997). These widely expressed chemoattractant cytokines play an important role in regulating cell traffic processes (Bromley et al., 2008). Subsequently their role is extended to other pathological and physiological conditions including angiogenesis, hematopoiesis, atherosclerosis, and cancer (Romagnani et al., 2004). According to the conserved cysteine residues from N-terminal, chemokine receptors can be classified into four subfamilies: (CXC, CC, CX3C, and C). Furthermore, the conserved ELR-motif is shared by some members of CXC-chemokines which exert angiogenic effects while those lacking ELR-motif are angiostatic (Strieter et al., 2005). Chemokine receptors can also be classified based on their functions: homeostatic and inflammatory, or both. The constitutively expressed homeostatic chemokines play a key role not only in development but also in maintenance of immune systems, whereas inflammatory chemokines are induced when the relevant cells are stimulated (Vandercappellen et al., 2008).

CXCR7 is a seven transmembrane-spanning receptor. It has been re-named to ACKR3, implying it belongs to the atypical chemokine receptors (ACKRs) family. Most ACKRs lack functional domains for G_i_ protein coupling and signaling (Bachelerie et al., 2014). The gene sequence of CXCR7 remains highly conserved among humans, dogs, mice, and rats (Libert et al., 1990). Originally it was cloned from a dog thyroid and named RDC-1 (Libert et al., 1989). In 2005, considering the similarity of its structure to CXC receptors, it was renamed CXCR7 according to chemokine receptor nomenclature (Balabanian et al., 2005; Graham et al., 2012). The gene of human CXCR7 is located on the region of 2q37.3 whereas in mice it is at 55.6 cM in the chromosome (Heesen et al., 1998). Although CXCR7 encodes two exons, only the last exon is the solely translated coding region (Broberg et al., 2002). Originally CXCR7 was considered as a receptor for calcitonin gene-related peptide (CGRP1) and vasoactive intestinal peptide (VIP) but studies have not been able to prove this (Cook et al., 1992; McLatchie et al., 1998). Unlike typical GPCRs, CXCR7 was shown not coupled with G_i_ proteins. Most chemokine receptors share a conserved motif DRYLAIV at the N-terminus of the second intracellular loop which is critical for calcium signaling and Gi protein coupling. The CXCR7 sequence is altered to DRYLSIT (A to S and V to T) and this structural difference was considered the reason on why CXCR7 is unable to induce cell signaling transduction through Gi proteins (Graham et al., 2012). At this point, how CXCR7 mediates activation of intracellular pathways remains controversial. Some studies have indicated that CXCR7 is a scavenger or decoy receptor which does not couple to Gi-proteins (Betterman and Harvey, 2014; Klein et al., 2014). Other evidence suggests that CXCR7 physically associated with CXCR4, leading to a change of CXCR4 signaling and cellular functions (Levoye et al., 2009; Décaillot et al., 2011). In addition, a few studies demonstrate that CXCR7 can independently induce cell signaling via β-arrestin in certain cell lines (Rajagopal et al., 2010; Chen et al., 2015). Interestingly, as of now, only in astrocytes, it was shown that CXCR7 was able to couple with Gi/o proteins and induced cell signaling (Odemis et al., 2012). None of these studies is conclusive, however. This is particular true, because CXCR7 often are co-expressed with CXCR4 in the same cells and full deletion of either receptor *in vitro* or *in vivo* are not warranted. Thus, many of the cellular functions including cell migration and proliferation ascribed to CXCR7 need to be further rigorously confirmed.

This review focuses on discoveries related to endogenous and exogenous ligands for CXCR7. Some of them exclusively bind to CXCR7 while others have additional target receptors apart from CXCR7. Although all these ligands can bind to CXCR7, they can trigger totally different functional outcomes, with additional complexity of tissue-biased signaling as well. These aspects of consideration may explain why the contradicting opinions of CXCR7 exist. We will also emphasize the main disease relevance of CXCR7 and the therapeutic prospects CXCR7 may offer.

## Pharmacological Ligands of CXCR7

### Endogenous Ligands for CXCR7

#### SDF-1(CXCL12)

Stromal-cell derived factor (SDF-1), also known as CXCL12, is a pleiotropic chemokine (Lataillade et al., 2004) widely expressed among different organs including bone marrow, liver, heart, kidney, thymus, stomach, lymph nodes, pituitary gland, and brain (Juarez et al., 2004). Beyond these, CXCL12 may be highly induced under certain pathological conditions including ischemia, inflammation, hypoxia, cancer, and autoimmune diseases (Li and Ransohoff, 2009; Karin, 2010). At first, CXCL12 was regarded as a soluble pre-B-cell growth stimulating factor (PBSF) which facilitates progenitor proliferation of bone marrow B cells (Nagasawa et al., 1996), and later on its interaction with CXCR4 was discovered (Bleul et al., 1996). More recently, research found that CXCL12 can take part in homing of progenitor leukocytes into the bone marrow microenvironment and adaptive immune processes (Ishii et al., 1999; Nanki and Lipsky, 2000).

Initially, CXCR4 was thought to be the exclusive receptor for CXCL12. Later on, CXCR7 was found to be a second receptor for CXCL12 at a 10-fold higher binding affinity compared to CXCR4 (Balabanian et al., 2005). The signaling activity of CXCL12 is crucial in neural, vascular, and cardiac development and craniofacial organogenesis. When binding to CXCR4, CXCL12 changes its three-dimensional conformation and initiates exchanging from GTP to GDP and dissociates into α- and βγ-subunits, then activates several cell signaling pathways (Bajetto et al., 2001). Through modulating adenylyl cyclase activity, the α_i_ subunits inhibit cAMP formation; and the βγ subunits can activate PLC-β, which in turn hydrolyzes PIP2 (phosphatidylinositol 4, 5-bisphosphate) facilitating the production of DAG (diacylglycerol) and IP3 (inositol) 1, 4, 5 triphosphate. These secondary messengers lead to release of Ca^2+^ from ER and activate protein kinase C. CXCL12 can activate PI3K (phosphoinositide 3-kinase)/Akt, IP3, and MAPK (mitogen activated protein kinase) through CXCR4 to regulate cell survival, chemotaxis, and proliferation. In addition, CXCL12 also activates JNK and p38 to control cell survival (Pan et al., 2013; Lin et al., 2014; Teng et al., 2016). In our lab we found that CXCR7 was expressed in macrophage-positive area of aortic atheroma of ApoE-null mice, but not in healthy arteries. Consistent with this, we found that during monocyte-to-macrophage differentiation process, CXCR7 was dramatically induced both at mRNA and protein levels. This CXCR7 induction prompted a CXCL12 signaling switch from pro-survival pathways (AKT and ERK1/2) to pro-inflammatory pathways (p38 and JNK), leading to increased macrophage phagocytosis (Wanshu et al., 2013).

At one point CXCR7 was regarded as a scavenger or decoy receptor for CXCL12. The original *in vivo* findings in zebrafish indicate that CXCR7 functions primarily by sequestering CXCL12, leading to a CXCL12 gradient formation (Boldajipour et al., 2008). It was also shown in mice that CXCR7 appears to function as a scavenger receptor for CXCL12 to limit B cells in the splenic marginal zone (Wang et al., 2012). Consistent with this, although binding with CXCR7, CXCL12 is not able to mediate calcium mobilization (Zabel et al., 2009). Besides, β-arrestin is more likely to be activated by CXCL12 (Rajagopal et al., 2010). Sometimes CXCR4 and CXCR7 form heterodimers, in which case the conformation of CXCR4/G-protein complexes are changed by CXCR7 and thus its signaling capacity will be blocked (Levoye et al., 2009). It should be noted, however, that such a negative impact of CXCR7 on CXCR4 was only observed on cell lines with ectopically over-expressed receptors and it remains to be determined whether the same is true on the native CXCR4 and CXCR7 receptors. The formation of CXCR4-CXCR7 heterodimers can also enhance CXCL12-induced intracellular Ca^2+^ mobilization and ERK1/2 activation (Sierro et al., 2007). Through activation by CXCL12, the CXCR4–CXCR7 complex increases cell signaling by recruiting β-arrestin, including the ERK1/2 and p-38 pathways (Décaillot et al., 2011; Heinrich et al., 2012). Apart from these, other studies found that CXCR7 can independently mediate CXCL12-induced AKT and ERK activation via G-protein (Odemis et al., 2012) or by β-arrestin (Gravel et al., 2010; Rajagopal et al., 2010).

#### I-TAC (CXCL11)

IFN-inducible T cell α-chemoattractant (I-TAC), also termed CXCL11, is mainly expressed in the pancreas, peripheral blood leukocytes, thymus, liver, spleen, and lung. To a lesser degree, it is expressed in the prostate, placenta, and intestine (Cole et al., 1998). Just like CXCL12, CXCL11, binds to two chemokine receptors CXCR3 and CXCR7. Interferons have the ability to induce CXCL11 production among several cell lines including leukocytes, endothelial cells, and fibroblasts. The level of CXCL11 is up-regulated during an infection or cancer process. There are two variants of CXCR3: CXCR3-A and CXCR3-B. When binding with CXCR3-A or CXCR7, CXCL11 was found to promote cell proliferative signaling. In contrast, it inhibits the effect on cell growth while binding to CXCR3-B (Lasagni et al., 2003). The role of CXCR3 in metastasis seems far more complex because of the two isoforms CXCR3-A and CXCR3-B. Similar to CXCR4, CXCR3-A activates Gi protein and regulates its metastatic effects (Wu et al., 2012). In contrast, CXCR3-B recruits Gs instead of Gi and inhibits metastatic signals. Both high expression of CXCR3-A and downregulation of inhibitory signals via CXCR3-B contribute to prostate cancer cell migration and invasion. This implies that CXCL11 may bind to CXCR3-A and activate its downstream cascades and also suppress the expression level of CXCR3-B. Although no papers clearly mentioned interactions between CXCR3-A/B and CXCR7, such a possibility of a crosstalk between CXCR3-A/B and CXCR7 in tumor migration and invasion process remains to be determined. Similar to CXCL12, when binding with CXCR7, CXCL11 is not able to induce calcium signaling or activate ERK1/2 or AKT (Proost et al., 2007). However, some studies have demonstrated that CXCL11 can promote ERK and AKT phosphorylations in CXCR4^+^CXCR7^+^CXCR3^-^ cell lines (Tarnowski et al., 2010b). The contradictory results may have been because of the different cell lines used (tissue specific biased signaling). Furthermore, binding of CXCL11 to CXCR7 also recruits β-arrestin-2 which indicates that subsequent signaling is regulated by β-arrestin. Further studies of CXCL11 and CXCR7 interactions are needed to clarify these issues.

#### MIF (Macrophage Inhibitory Factor)

Macrophage inhibitory factor acts as a pro-inflammatory cytokine with chemokine-like functions which regulate innate immunity. Originally, it was regarded as a T-cell derived factor which plays a role in inhibiting macrophage migration (David, 1966). It is widely expressed by several cells including eosinophils (Rossi et al., 1998), endothelial cells (Nishihira et al., 1998), epithelial cells (Imamura et al., 1996), lymphocytes (Bacher et al., 1996), and macrophages (Calandra, 1994). Under the dysregulated conditions, MIF participates in a series of diseases including rheumatoid arthritis, systemic lupus erythematosus, sepsis, glomerulonephritis, atherosclerosis, and cancer (Morand et al., 2006; Lang et al., 2015; Zwiech, 2015; Xiao et al., 2016).

Several receptors have been shown to mediate MIF functions. The first is CD (cluster of differentiation) 74. CD74 is a single-pass type II transmembrane protein which is also known as the plasma membrane form of the major histocompatibility class II invariant chain (Ii) (Leng et al., 2003). Binding to CD74 mainly mediates survival and proliferative functions of MIF on immune and tumor cells. With the help of co-receptor CD44, MIF can induce signaling through CD74 to activate tyrosine kinases and the PI3K/AKT pathway (Shi et al., 2006; Lue et al., 2007). There is also evidence that MIF interacts with CD74 and subsequently activates ERK1/2 pathways (Shi et al., 2006). Apart from CD74, MIF is also identified as a non-cognate ligand for two chemokine receptors, CXCR2 and CXCR4, which regulate cell signaling through their cognate ligands CXCL1/CXCL8 and CXCL12, respectively. CXCR2 and CD74 can form heterodimers which is very important in MIF-regulated atherogenic leukocyte recruitment (Bernhagen et al., 2007). Besides, CXCR4/CD74 complexes are able to activate the AKT pathway through interaction with MIF (Schwartz et al., 2009). Interestingly, signaling pathways and pathogenic effects which are regulated by MIF have been found recently to be linked with CXCR7. Both MIF-CXCR4 and MIF-CXCR7 axis play an important role in Rhabdomyosarcoma tumor cell migration. Furthermore, in platelets, MIF is able to activate AKT signaling pathway through CXCR7 to limit apoptosis (Tarnowski et al., 2010a; Chatterjee et al., 2014). In addition to these, MIF is also found to promote CXCR7 internalization, induce B-cell chemotaxis, and ERK1/2 activation (Alampour-Rajabi et al., 2015). These discoveries indicate that CXCR7 is a new receptor for MIF.

#### ADM (Adrenomedullin)

As a peptide, ADM was first identified as a potent vasodilator (Kitamura et al., 1993). ADM is a mitogenic hormone with 52 amino acids which plays a crucial role in lymphatic vascular and cardiac development (Caron and Smithies, 2001; Dunworth et al., 2008; Fritz-Six et al., 2008). It can activate cognate receptors consisting of CLR (calcitonin receptor-like receptor) and RAMP (receptor activity-modifying protein). There is also evidence that CXCR7 is able to bind to ADM with a *K*_d_ of 1.9 × 10^-7^ M (Kapas and Clark, 1995). A recent study proved that the genetically overexpressed ADM ligand caused hypertrophic heart development during embryogenesis in Adm^hi/hi^ mice (Wetzel-Strong et al., 2014). In keeping with the crucial role of ADM in promoting lymph-angiogenesis, it was discovered that CXCR7^-/-^ presented lymphatic vascular defects. Based on their findings, the investigators suggested that CXCR7 acts as a decoy receptor for ADM in controlling cardiac and lymphatic development (Klein et al., 2014), a new mechanism that may explain the earlier findings by others in the field (Dunworth et al., 2008; Fritz-Six et al., 2008; Karpinich et al., 2011; Hoopes et al., 2012). Whether or not ADM can induce any signaling transduction through CXCR7 needs to be determined by further studies.

#### BAM22 (Bovine Adrenal Medulla 22)

As one of the cleaved products of proenkephalin A, BAM22 was initially isolated from the bovine adrenal medulla (Mizuno et al., 1980; Dores et al., 1990). In mammals, BAM22 is widely distributed in the central nervous system (Khachaturian and Lewis, 1983; Pittius et al., 1984). BAM22 has the classical opioid YGGFM motif and exerts both opioid and non-opioid actions (Boersma et al., 1994). Through activating three major opioid receptors, μ-, δ-, and κ- (Garzon et al., 1983; Quirion and Weiss, 1983; Lembo et al., 2002), BAM22 not only inhibits contractions of ileum (Davis et al., 1990), vas deferens (Sánchez-Blázquez and Garzón, 1985), and bladder (Dray et al., 1985), but also induces nociceptive response (Höllt et al., 1982), which is sensitive to naloxone, a medication used to block the effects of opioids. It is quite important that as a unique endogenous peptide, BAM22 exhibits a high affinity when binding to the human Mas oncogene-related gene (Mrg) receptors, which are restricted to small-diameter DRG neurons in humans and rodents (Dong et al., 2001; Lembo et al., 2002). Research has proven that altered BAM22 expression in DRG or spinal dorsal horn in complete Freund’s adjuvant (CFA)-induced chronic pain and morphine tolerance which suggested that BAM22 participates in the nociceptive process (Cai et al., 2007; Chen et al., 2008). Recently it was found that CXCR7 is a high-affinity receptor for BAM22 which promoted glucocorticoid secretion. BAM22 can recruit both β-arrestin1 and β-arrestin2 at nanomolar concentrations after binding with CXCR7. Consistent with previous studies of CXCR7, BAM22 elicited neither a cyclic AMP (cAMP) nor a calcium response in H295R cells and human 293T cells, both of which express high levels of CXCR7. Intriguingly, the interaction between BAM22 and CXCR7 is also insensitive to naloxone (Ikeda et al., 2013). Further studies on possible cell signaling transduction between BAM22 and CXCR7 need to be explored.

#### vCCL2/vMIP-II

Viruses are grouped into different families such as herpesviruses, retroviruses, and poxviruses. These pathogens encode chemokine-binding proteins, chemokine receptors, and chemokine analogs that hijack cellular chemokine receptors (Alcami, 2003; Vischer et al., 2014). Human herpesvirus 8 (HHV-8), also called Kaposi’s sarcoma-associated herpesvirus (KSHV), is a good example. HHV-8 can lead to Kaposi’s sarcoma (KS) which is closely related to immunodeficiency. It can also cause two rare proliferative disorders-primary effusion lymphoma (PEL) and multicentric Castleman disease (MCD).

vCCL2, also known as vMIP-II, is a viral CC chemokine encoded by HHV-8. This chemokine was first discovered from the HHV-8 genome which was isolated from a KS biopsy (Nicholas et al., 1997). Three of the ORFs in HHV-8 were predicted to encode the chemokine homologs including vCCL1 (vMIP-I), vCCL2 (vMIP-II), and vCCL3 (vMIP-III), respectively, plus one CXC chemokine receptor homolog ORF74 (Moore et al., 1996). vCCL2 yields in a mature format as a 70-aa chemokine (7.9 kDa), and its 94-aa precursor endows a 23-aa N-terminal signal peptide and a C-terminal arginine (Moore et al., 1996; Kledal et al., 1997).

As an active chemokine, vCCL2 is able to bind with four classes of receptors. They include CC family: CCR1, CCR2, CCR3, CCR5, CCR8, CCR10; XC family: XCR1; CX3C family: CX3CR1; and CXC family: CXCR4 (Lüttichau et al., 2007; Qin et al., 2015; Szpakowska and Chevigné, 2015). In addition, it has the ability to downregulate the activity of ORF74 (Geras-Raaka et al., 1998). In most cases, vCCL2 is regarded as an antagonist chemokine whereas it can also act as agonist toward CCR3 and CCR8 (Szpakowska and Chevigné, 2015). One thing noteworthy is that recently vCCL2 was found to act as a partial agonist of CXCR7 which can induce β-arrestin recruitment to the receptor. vCCL2 triggers MAP kinase and PI3K/AKT signaling through other chemokine receptors which can be reduced by CXCR7 expression (Szpakowska et al., 2016). This study provided new insights into the interaction between viral chemokines and ACKRs like CXCR7.

### Exogenous/Synthetic Ligands for CXCR7

#### VUF11207 and VUF11403

From a styrene-amide scaffold, 24 derivatives were synthesized. These CXCR7 ligands were evaluated with pK_i_ values ranging from 5.3 to 8.1. With the help of SAR studies, two key compounds, VUF11207 and VUF11403, were found to have high affinity with CXCR7. These two CXCR7 agonists are able to recruit β-arrestin2 and reduce CXCR7 surface expression. Taken together, these two ligands have great value in CXCR7 study (Wijtmans et al., 2012).

#### AMD3100

AMD3100 is a small molecule which belongs to the bicyclam family. Initially AMD3100 was found to have antiretroviral effects and later on was shown to bind with CXCR4. Through interaction with CXCR4, AMD3100 shows strong and selective inhibitory effects of X4-tropic HIV replication *in vitro* (De Clercq, 2005). *In vivo*, the anti-HIV effect of AMD3100 was proved in the immunodeficiency (SCID)-Hu Thy/Liv mouse model (Datema et al., 1996). Based on these properties, AMD3100 is widely used as a tool for demonstrating the interaction between CXCL12 and CXCR4. As CXCR4 plays an important role during the hematopoietic stem cell homing process, singly administrated high doses of AMD3100 caused a huge release of these cells into peripheral blood. Thus, AMD3100 and its derivatives are undergoing testing for treatment of cancer (De Clercq, 2005).

Using a homodimeric receptor BRET sensor, researchers also found AMD3100 to be a ligand for CXCR7. Different from the antagonism effect on CXCR4, AMD3100 enhances CXCL12 binding with CXCR7. In addition, AMD3100 alone is able to recruit β-arrestin to CXCR7 but inhibits recruiting to CXCR4. Thus, AMD3100 is regarded as an agonist for CXCR7, albeit at relatively high concentrations (≥10 μM) (Kalatskaya et al., 2009).

#### TC14012

TC14012 was modified from the parental entity TC140 which is a peptidomimetic derived from horseshoe crab polyphemusin and described as an inverse agonist of CXCR4 (Tamamura et al., 1998; Trent et al., 2003). TC14012, as a serum-stable compound, is able to recruit β-arrestin 2 to CXCR7 (Tamamura et al., 2001). Compared to AMD3100, TC14012 showed much higher potency with CXCR7 (EC_50_ of 350 nM for TC14012 vs. 140 μM for AMD3100) and only one log unit weaker than the natural ligand CXCL12 (35 nM) (Gravel et al., 2010). Significant similarities were evident between the binding mode of TC14012 to CXCR7 and CVX15 to CXCR4 (Wu et al., 2010), which provided new insight into the interaction between TC14012 and CXCR7. Thus, TC14012 can be a helpful tool for studying the biology and pharmacology of CXCR7; however, it should be noted that TC14012 has antagonistic activity on CXCR4 as well, which complicates data interpretation when it is used for mechanistic study.

#### CCX771

CCX771 is a selective small molecule agonist and binds to human CXCR7 with an IC_50_ of 4.1 nM. It was patented by ChemoCentryx and has not been commercially available (Zabel et al., 2009). It has been reported that CCX771 is highly selective for CXCR7 and had no effect on CXCL12 binding to CXCR4 in NC-37 tumor cells (Zabel et al., 2009). At present, CCX771 is often used in researches for studying the role of CXCR7 in different cell lines and animal models. For instance, CCX771 was able to inhibit tumor growth, lung metastasis, and tumor angiogenesis *in vivo*. This helped researchers unveiling that CXCL12-CXCR7 autocrine loop affects tumor endothelial cells proangiogenic properties (Yamada et al., 2015). However, it remains uncertain whether many of the observed effects of CCX771 treatment is due to its agonistic activity on CXCR7 or its antagonistic effect on endogenous agonists such as CXCL12.

#### Other Synthetic Ligands for CXCR7

Although no specific name was offered, Boehm et al recently reported a macrocyclic peptide-peptoid hybrid molecule, which binds to CXCR7 with high affinity (*K*_i_ < 100 nM) and measurable passive permeability (*P*_app_ > 5 × 10^-6^ cm/s). The bioactive peptide 25 (*K*_i_ = 9 nM) achieved oral bioavailability of 18% in rats, which was commensurate with the observed plasma clearance values upon intravenous administration (Boehm et al., 2017). In addition, FC313, a cyclic pentapeptide ligand for CXCR7, which is modified at the I-Pro position with a bulky hydrophobic side chain, exhibited an improved bioactivity toward CXCR7 (Sekiguchi et al., 2018). Most recently, Ameti et al. (2018) reported a chimeric chemokine, which selectively binds to CXCR7. This chimera is composed of the N-terminus of CXCL11 and the main body and C-terminus of CXCL12 and selectively interacts with CXCR7 with high affinity, while not interfering with binding of CXCL11 and CXCL12 to their cognate receptors (Ameti et al., 2018). We believe that all these newly generated ligands will be valuable pharmacologic tools for the study of CXCR7.

## Major Disease Relevance for CXCR7

### Cancers

The expression levels of CXCL12 and its receptors were described in several types of solid tumors and tumor cells, including lung, prostate, breast, and pancreatic cancers (Koshiba et al., 2000; Wang et al., 2008; Wu W. et al., 2015; Wu Y.-C. et al., 2015). The particular microenvironment of tumors controls CXCR7 expression. For instance, under hypoxic conditions, the transcription level of CXCR7 in human microvascular endothelial cells and the translation level of CXCR7 in glioma cell lines were increased (Schutyser et al., 2007; Esencay et al., 2013). Consistently, results showed that hypoxia-inducible factor 1 alpha leads to up-regulated CXCR7 transcript in mesenchymal stem cells (Liu et al., 2010). Tumor suppressor genes are silenced when DNA is methylated, thus there is evidence that the transcriptional level of CXCR7 is regulated by a cancer 1 (HIC1) tumor suppressor (Van Rechem et al., 2009). For example, in prostate cancer cells, HIC1 negatively regulated the CXCR7 promoter (Zheng et al., 2013). The expression level of CXCR7 is controlled by miRNA-430 in zebrafish which suggests that the overexpression of CXCR7 is caused by a lack of miRNA-mediated regulation (Staton et al., 2011). Consistent with this, downregulation of miRNA-430 induced a high expression level of CXCR7 in a bladder cancer cell line (Liu et al., 2013). In addition, the restoration of an important tumor-suppressive miRNA named miRNA-101, inhibited CXCR7 protein synthesis in normal hepatocyte-derived cell lines, different hepatocellular carcinoma cell lines, primary hepatocytes and xenograft mice models (Wang et al., 2014). Furthermore, Liu et al. (2017) recently reported that in oral tongue squamous cell carcinoma, CXC chemokine-7 produced an inhibitory effect in cell growth and migration, which is mediated by epithelial to mesenchymal transition (EMT) signaling pathway. This implicates CXCR7 as a promising biomarker for chemokine receptor-based drug development (Liu et al., 2017).

Many tumor cells have the ability to produce CXCL12, whose extracellular bioavailability can be modulated by the cell-surface expressed CXCR4 and CXCR7. This was proved in *in vivo* imaging which showed that tumor cells expressing CXCR7 decreased the concentration of CXCL12 in the primary tumor microenvironment (Luker et al., 2012). Monomeric or dimeric forms of CXCL12 can make significant differences. Among different types of cancers, the dimeric form of CXCL12 can produce opposite effects. For instance, the dimeric CXCL12 is more potent than monomeric CXCL12 in promoting β-arrestin 2 recruitment and chemoattractive activity in a model of human breast cancer (Ray et al., 2012). In contrast, when it moved to a human colon carcinoma cell line, CXCL12 induced calcium mobilization, β-arrestin 2 recruitment, and cell migration from monomeric form whereas it only weakly induced chemotaxis and β-arrestin 2 recruitment (Drury et al., 2011). The differences between monomeric and dimeric CXCL12 forms are still not clear enough for unquestionable conclusions.

Depending on different tumor types, CXCR7 may or may not co-express with CXCR4. These two receptors can be distinctively expressed in glioma and breast cancer (Hattermann et al., 2010; Luker et al., 2012). In contrast, CXCR7 and CXCR4 often co-express in human pancreatic cancer tissues (Heinrich et al., 2012). The single expression of these two receptors on certain cell populations restricts their ability for paracrine regulation. By comparison, co-expression of these two receptors on the same cell population made the direct interaction between CXCR4 and CXCR7 and controlled signaling pathways reciprocally possible.

The CXCL12-mediated chemotactic function was changed when both CXCR4 and CXCR7 expressed on the same cell line. For instance, when CXCR7 was transfected into breast cancer cell line MDA-MB-231, the CXCL12-induced chemotactic function was increased (Décaillot et al., 2011). Also, under the condition of upregulated CXCR4 expression, CXCR7 enhanced cell chemotaxis in response to CXCL12 in rat mammary adenocarcinoma cell line MTLn3 (Hernandez et al., 2011). However, it was also shown that CXCR7 limited chemotaxis effects through the interaction of CXCR4 and CXCL12 in human neuroblastoma cell lines (Liberman et al., 2012).

The key element for promoting angiogenesis and malignant cell migration is the distribution of endothelial progenitor cells (EPCs), which is controlled by trans-endothelial migration. Since CXCR7 is highly expressed on EPCs, it determines the survival effect of CXCL12 on EPCs. In addition, CXCR7 also influences these CXCL12-regulated processes including trans-endothelial migration, proliferation, adhesion, and tube formation of EPCs (Dai et al., 2011). The increased expression of CXCR7 is beneficial for angiogenesis in cancers which indicates the key role of CXCR7 in controlling the angiogenic process. Under hypoxic conditions, CXCL8 and VEGF were highly increased in the tumor microenvironment, which amplifies CXCR7 expression through the positive feedback mechanism (Singh and Lokeshwar, 2011). In breast cancer, CXCR7 activation promotes primary tumor growth through increasing VEGF production and microvessel density (Hernandez et al., 2011). When applied to osteosarcoma and associated lung metastasis, the evidence that CXCR7 is significantly expressed on tumor-associated vessels confirmed its critical role in the metastatic process (Goguet-Surmenian et al., 2013). In hepatocellular carcinoma, CXCR7 is able to enhance endothelial cell proliferation, migration, and VEGF production which mediate angiogenesis and tumor growth (Zheng et al., 2010). In a nut shell, CXCR7 seems very important for angiogenesis and metastasis in tumor cells.

Despite the accumulating evidence, whether the binding of CXCL12 to CXCR7 can directly induce cell migration is still in debate, so in non-small lung cancer cells, CXCR7 has not been conclusively implicated in CXCL12-regulated behavior (Choi et al., 2014). However, the CXCL12 effects regulated through CXCR4 for promoting metastasis can be affected by CXCR7. Under certain conditions, CXCR7 has the ability to impair CXCR4-regulated effects. In breast cancer derived from an immune-deficient mouse model, CXCR7 prevented tumor cell invasion and spontaneous lung metastasis formation (Hernandez et al., 2011). Also, the interaction between CXCL12 and CXCR7 did influence CXCR4-mediated trans-endothelial migration of human tumor cells (Zabel et al., 2009, 2011). On the contrary, the activation of CXCR7 was able to promote metastasis in the breast cancer model (Miao et al., 2007). The role of CXCR7 in tumor migration is still not conclusive and further studies on its role with CXCR4 are needed.

The discovery of CXCR7 has provided human beings a viable target for anti-tumor and anti-metastatic drugs. Using the model of mice engrafted with breast and lung cancer cell lines, inhibiting CXCR7 with believed antagonists showed that CXCR7 is able to promote tumor growth (Miao et al., 2007). The main purpose for developing CXCR7 antagonists is to decrease the spreading of tumor cells, their metastasis, and angiogenesis. As an example, CCX771, a synthetic CXCR7 antagonist, showed high potential of inhibiting trans-endothelial migration as compared to AMD3100, a CXCR4 antagonist. However, in a lymphoblastic leukemia model, CCX771 also recruited β-arrestin to CXCR7 (Zabel et al., 2009). Thus, whether CCX771 should be considered as an antagonist or agonist, needs to be further explored. This is an important question when considering CXCR7 as a target for cancer treatment. On the other hand, it was shown that both CXCR4 and CXCR7 responded to CXCL12 to greatly increase human lymphoma cells’ migration, indicating that CXCR7 might be an efficient target for cancer treatment (Zabel et al., 2011).

Since the expression of CXCR7 may be able to direct hematopoietic stem cells (HSCs) to the niches which sustain their migration capacity, CXCR7 is a potential regulatory target for HSCs mobilization-inducing agent development. This is supported by an *in vitro* study indicating that although CXCR7 is not an intrinsic signaling receptor for CXCL12 on CD34^+^ HSCs, its blocking can be useful for therapeutic interference with CXCR4-mediated activation of integrins and cell adhesion (Hartmann et al., 2008). In addition, a recent *in vivo* study provided further evidence indicating that CXCR7 contributes to homing of acute myeloid leukemia and normal CD34^+^ progenitor cells to the bone marrow and spleen of NOD/SCID mice (Melo et al., 2018); however, further mechanistic study is needed to fully understand the role of CXCR7 in HSCs. Although the ability of CXCR7 to regulate the BMSC niche is still in debate, studying CXCR7 antagonists is a hot spot among HSCs mobilizers because this may provide patients with an alternative treatment when other mobilization protocols fail (To et al., 2011).

The structural model for CXCR7 is very helpful for elucidating the pharmacology and potential therapeutic utility of CXCR7 antagonists. Unfortunately, there are no detailed structures available at present and only a limited number of ligands for CXCR7 have been reported (Kalatskaya et al., 2009; Wijtmans et al., 2012; Montpas et al., 2015). Therefore, approaches such as virtual screening and GPCR homology modeling which have been used in the previous studies of CXCR4 can be promising tools for new CXCR7 ligand identification (Yoshikawa et al., 2013). A number of pharmacological studies focus on the small molecules of CXCR7 antagonists. These antagonists endow reasonable affinities but researchers lack structural information (Burns, 2006; Zabel et al., 2009; Hattermann et al., 2010; Cruz-Orengo et al., 2011). The synthetic, modeling, and pharmacological effect on small molecules targeting CXCR7 was described in a recent report (Wijtmans et al., 2012).

The molecules which can block CXCR4 and CXCR7 simultaneously, represent an ideal pharmacological approach because both receptors are involved in cancer malignancy and GBM angiogenesis (Duda et al., 2011). However, the current available data governing the binding of these two receptors seems rather complex. Some antagonists did not bind to CXCR4 or CXCR7 exclusively, but bound to the other one, too. And even when they didn’t act as antagonist, partial agonist activity did show up. This happened on AMD3100, a CXCR4 antagonist which may also act as a CXCR7 partial agonist (Kalatskaya et al., 2009). In addition, the expression levels of CXCR4 were down-regulated through CXCR7 agonists which selectively activated β-arrestin (Uto-Konomi et al., 2013). These complex biological responses may be due to cell type specific biased signaling. For GBM, CXCR4 was mainly expressed in CSCs whereas CXCR7 is mainly distributed in differentiated cells and endothelia (Hattermann et al., 2010; Gatti et al., 2013). In other situations these two receptors are often co-expressed and could potentially form heterodimers. Thus the study of ligands which can interact with both CXCR4 and CXCR7 must be evaluated in specific cell type while considering agonist/antagonist properties of the molecule. Blocking both CXCR4 and CXCR7 through influencing their shared ligands will help researchers better understand the interaction mode between the ligands and the CXCR4/CXCR7 receptors. Synthetic compounds derived from the family of chalcones, have high affinity binding with CXCL12 and can prevent CXCL12 from interacting with CXCR4 and CXCR7. Such a process is able to inhibit inflammatory responses in eosinophils (Hachet-Haas et al., 2008). In addition, there is an RNA oligonucleotide named NOX-A12 which can bind and neutralize CXCL12 with high affinity (Liang et al., 2007). Because of the antitumor activity, NOX-A12 is now clinically available for treatment of leukemia and multiple myeloma. It is also noteworthy that, in an *in vivo* model of GBM, NOX-A12 was effective in inhibiting or delaying recurrences following irradiation (Liu et al., 2014).

### Cardiovascular Diseases

CXCR7 is expressed in the developing heart and associated with defects in the cardiovascular system. Knocking out *CXCR7* gene can lead to a phenotype of hypertrophy including thickened pulmonary, aortic valves, and partially overridden aortas (Sierro et al., 2007; Gerrits et al., 2008; Yu et al., 2011). These abnormalities may have a relationship with disturbed endothelial cell migration because those hypertrophic defects were reproduced in endothelial cell specific CXCR7 knockout mice (Sierro et al., 2007). CXCR7 was regarded as a scavenger receptor for CXCL12 in heart valves, so one proposed hypothesis is that CXCR7 prevents the over-interaction between CXCL12 and CXCR4 through sequestering CXCL12 during heart development, which could lead to hyperplasia (Naumann et al., 2010). However, at this point, we could not rule out the possibility that CXCR7 may independently transduce cell signaling in some vascular cells. On this aspect, CXCL12-CXCR4 axis is credited with controlling the migration and proliferation of EPCs whereas CXCL12-CXCR7 mainly maintains the survival of EPCs and promotes these cells adhering to endothelial cells (Yan et al., 2012). It is also important to note that, CXCR7 can transduce cell signaling through β-arrestin to promote the migration of vascular smooth muscle cells (Rajagopal et al., 2010), strengthening the viewpoint of the importance of possible biased signaling on CXCR7. The formation of a CXCR4–CXCR7 heterodimer indicates that these two receptors may have mutual functions during angiogenesis. Either deletion of CXCR4 or CXCR7 leads to ventricular septum defects, showing that these two receptors are potential new intervention targets for studying certain cardiovascular diseases (Zou et al., 1998; Sierro et al., 2007). Again, it should be noted that whether the natural CXCR4 and CXCR7 receptors form heterodimers needs to be further investigated, since such heterodimers having enhanced responses to CXCL12 was observed only in HEK293 cells with overexpressed CXCR4 and CXCR7 (Sierro et al., 2007).

The pivotal role of CXCL12 in the cardiovascular system makes itself and its receptors a hotspot of cardiovascular disease research. During the past few years, scientists focused on the CXCL12-CXCR4 axis in myocardial infarction and heart ischemia whereas very few studies mentioned the function of CXCR7. Of the few studies available, one study discovered that CXCL12-β protects cardiac cells via CXCR7 (Zhao et al., 2013) and evidence implied that CXCR7 signaling is able to take part in the regeneration process after myocardial infarction (Sierro et al., 2007; Yan et al., 2012). In addition, it was found that lack of endothelial CXCR7 impairs vascular homeostasis and cardiac remodeling after myocardial infarction. This indicated that CXCR7 might be a potential therapeutic target for certain cardiovascular diseases, such as restenosis (Hao et al., 2017). CXCR7 also produces beneficial effects on angiogenic function of EPCs, which are mediated predominantly by a protein kinase B/glycogen synthase kinase-3β/Fyn pathway via increased activity of Nrf2 (Dai et al., 2017). Further, it seems that CXCR7 expression is also critical for limiting endothelial-to-mesenchymal transition and pulmonary fibrosis (Guan and Zhou, 2017). Since we recently reported that during monocyte-to-macrophage differentiation, CXCR7 is dramatically induced and mediates CXCL12 signaling independent of CXCR4, which leads to increased macrophage phagocytosis (Ma et al., 2013). Furthermore, we reported that CXCR7 induction is suppressed by atorvastatin treatment, leading to decreased macrophagemigration in response to CXCL12 (Ma et al., 2014). Collectively, these findings highlight that CXCR7 may be a new therapeutic target for cardiovascular diseases, such as atherosclerosis.

## Conclusion and Perspectives

The axis of CXCL12-CXCR4 plays a very important role in physiology and pathology, and the discovery of CXCR7 as a new high affinity receptor for CXCL12 made this interaction system much more sophisticated. Previous studies mainly focused on the triangle relationship of CXCL12, CXCR4, and CXCR7, while other ligands for CXCR7 have been ignored. CXCR7 itself can play multiple roles with its endogenous and exogenous ligands. It may just act as a rheostat for certain ligands through scavenging them and not transducing any cell signaling pathways. It may also act as a co-factor to dimerize with other receptors in cell signaling transduction. In addition, CXCR7 may independently induce cell signaling transduction, either through G protein or β-arrestin, or other unknown transducer, depending on the cell types or different stages of cell differentiation. We are just discovering the tip of the iceberg of the biased signaling network system for CXCR7 and its ligands in various cell types. More interesting and vital connections are emerging from study of the massive iceberg that is CXCR7.

## Author Contributions

CW collected the original materials and wrote the first draft. WC and JS made significant changes on the scope and format of this review.

## Conflict of Interest Statement

The authors declare that the research was conducted in the absence of any commercial or financial relationships that could be construed as a potential conflict of interest.

## References

[B1] Alampour-Rajabi S. El Bounkari O. Rot A. Müller-Newen G. Bachelerie F. Gawaz M. (2015). MIF Interacts with CXCR7 to Promote Receptor Internalization, ERK1/2 and ZAP-70 Signaling, and Lymphocyte Chemotaxis. *FASEB J.* 29 4497–4511. 10.1096/fj.15-273904 26139098

[B2] Alcami A. (2003). Viral mimicry of cytokines, chemokines and their receptors. *Nat. Rev. Immunol.* 3 36–50. 10.1038/nri980 12511874

[B3] Ameti R. Melgrati S. Radice E. Cameroni E. Hub E. Thelen S. (2018). Characterization of a chimeric chemokine as a specific ligand for ACKR3. *J. Leukoc. Biol.* 10.1002/JLB.2MA1217-509R [Epub ahead of print]. 29601107

[B4] Bachelerie F. Ben-Baruch A. Burkhardt A. M. Combadiere C. Farber J. M. Graham G. J. (2014). International union of basic and clinical pharmacology. [Corrected]. LXXXIX. Update on the extended family of chemokine receptors and introducing a new nomenclature for atypical chemokine receptors. *Pharmacol. Rev.* 66 471–538. 10.1124/pr.113.007724 24218476 PMC3880466

[B5] Bacher M. Metz C. N. Calandra T. Mayer K. Chesney J. Lohoff M. (1996). An essential regulatory role for macrophage migration inhibitory factor in T-cell activation. *Proc*. *Natl. Acad. Sci. U.S.A.* 93 7849–7854. 10.1073/pnas.93.15.7849 PMC38837 8755565

[B6] Bajetto A. Bonavia R. Barbero S. Florio T. Schettini G. (2001). Chemokines and their receptors in the central nervous system. *Front*. *Neuroendocrinol.* 22 147–184. 10.1006/frne.2001.0214 11456467

[B7] Balabanian K. Lagane B. Infantino S. Chow K. Y. Harriague J. Moepps B. (2005). The chemokine SDF-1/CXCL12 binds to and signals through the orphan receptor RDC1 in T lymphocytes. *J. Biol. Chem.* 280 35760–35766. 10.1074/jbc.M508234200 16107333

[B8] Bernhagen J. Krohn R. Lue H. Gregory J. L. Zernecke A. Koenen R. R. (2007). MIF is a noncognate ligand of CXC chemokine receptors in inflammatory and atherogenic cell recruitment. *Nat*. *Med.* 13 587–596. 10.1038/nm1567 17435771

[B9] Betterman K. L. Harvey N. L. (2014). Decoys and cardiovascular development: CXCR7 and regulation of adrenomedullin signaling. *Dev. Cell* 30 528–540. 10.1016/j.devcel.2014.08.021 25203203

[B10] Bleul C. C. Farzan M. Choe H. Parolin C. Clark-Lewis I. Sodroski J. (1996). The lymphocyte chemoattractant SDF-1 is a ligand for LESTR/fusin and blocks HIV-1 Entry. *Nature* 382 829–833. 10.1038/382829a0 8752280

[B11] Boehm M. Beaumont K. Jones R. Kalgutkar A. S. Zhang L. Atkinson K. (2017). Discovery of potent and orally bioavailable macrocyclic peptide-peptoid hybrid CXCR7 modulators. *J*. *Med. Chem.* 60 9653–9663. 10.1021/acs.jmedchem.7b01028 29045152

[B12] Boersma C. J. C. Pool C. W. Van Heerikhuize J. J. Van Leeuwen F. W. (1994). Characterization of opioid binding sites in the neural and intermediate lobe of the rat pituitary gland by quantitative receptor autoradiography. *J*. *Neuroendocrinol.* 6 47–56. 10.1111/j.1365-2826.1994.tb00554.x 8025568

[B13] Boldajipour B. Mahabaleshwar H. Kardash E. Reichman-Fried M. Blaser H. Minina S. (2008). Control of chemokine-guided cell migration by ligand sequestration. *Cell* 132 463–473. 10.1016/j.cell.2007.12.034 18267076

[B14] Broberg K. Zhang M. Strömbeck B. Isaksson M. Nilsson M. Mertens F. (2002). Fusion of RDC1 with HMGA2 in lipomas as the result of chromosome aberrations involving 2q35-37 and 12q13-15. *Int*. *J. Oncol.* 21 321–326. 10.3892/ijo.21.2.321 12118328

[B15] Bromley S. K. Mempel T. R. Luster A. D. (2008). Orchestrating the orchestrators: chemokines in control of T cell traffic. *Nat*. *Immunol.* 9 970–980. 10.1038/ni.f.213 18711434

[B16] Burns J. M. (2006). A novel chemokine receptor for SDF-1 and I-TAC involved in cell survival, cell adhesion, and tumor development. *J. Exp. Med.* 203 2201–2213. 10.1084/jem.20052144 16940167 PMC2118398

[B17] Cai M. Chen T. Quirion R. Hong Y. (2007). The involvement of spinal bovine adrenal medulla 22-like peptide, the proenkephalin derivative, in modulation of nociceptive processing. *Eur. J. Neurosci.* 26 1128–1138. 10.1111/j.1460-9568.2007.05755.x 17767492

[B18] Calandra T. (1994). The macrophage is an important and previously unrecognized source of macrophage migration inhibitory factor. *J. Exp. Med.* 179 1895–1902. 10.1084/jem.179.6.1895 8195715 PMC2191507

[B19] Caron K. M. Smithies O. (2001). Extreme hydrops fetalis and cardiovascular abnormalities in mice lacking a functional adrenomedullin gene. *Proc*. *Natl. Acad. Sci. U.S.A.* 98 615–619. 10.2307/3054732 PMC14636 11149956

[B20] Chatterjee M. Borst O. Walker B. Fotinos A. Vogel S. Seizer P. (2014). Macrophage migration inhibitory factor limits activation-induced apoptosis of platelets via CXCR7-dependent Akt signaling. *Circ*. *Res.* 115 939–949. 10.1161/CIRCRESAHA.115.305171 25266363

[B21] Chen P. Liu Y. Hong Y. (2008). Effect of chronic administration of morphine on the expression of bovine adrenal medulla 22-like immunoreactivity in the spinal cord of rats. *Eur*. *J. Pharmacol.* 589 110–113. 10.1016/j.ejphar.2008.06.023 18577380

[B22] Chen Q. Zhang M. Li Y. Xu D. Wang Y. Song A. (2015). CXCR7 mediates neural progenitor cells migration to CXCL12 independent of CXCR4. *Stem Cells* 33 2574–2585. 10.1002/stem.2022 25833331 PMC4867224

[B23] Choi Y. H. Burdick M. D. Strieter B. A. Mehrad B. Strieter R. M. (2014). CXCR4, but Not CXCR7, discriminates metastatic behavior in non-small cell lung cancer cells. *Mol. Cancer Res.* 12 38–47. 10.1158/1541-7786.MCR-12-0334 24025971 PMC4262747

[B24] Cole K. E. Strick C. A. Paradis T. J. Ogborne K. T. Loetscher M. Gladue R. P. (1998). Interferon-inducible T Cell alpha chemoattractant (I-TAC): a novel non-ELR CXC chemokine with potent activity on activated t cells through selective high affinity binding to CXCR3.*J*. *Exp. Med.* 187 2009–2021. 10.1084/jem.187.12.2009 9625760 PMC2212354

[B25] Cook J. S. Wolsing D. H. Lameh J. Olson C. A. Correa P. E. Sadee W. (1992). Characterization of the RDC1 gene which encodes the canine homolog of a proposed human VIP receptor expression does not correlate with an increase in VIP binding sites. *FEBS Lett.* 300 149–152. 10.1016/0014-5793(92)80184-I 1373390

[B26] Cruz-Orengo L. Chen Y. Jr. Kim J. H. Dorsey D. Song S. K. Klein R. S. (2011). CXCR7 antagonism prevents axonal injury during experimental autoimmune encephalomyelitis as revealed by in vivo axial diffusivity. *J. Neuroinflammation* 8:170. 10.1186/1742-2094-8-170 22145790 PMC3305694

[B27] Dai X. Tan Y. Cai S. Xiong X. Wang L. Ye Q. (2011). The role of CXCR7 on the adhesion, proliferation and angiogenesis of endothelial progenitor cells. *J. Cell Mol. Med.* 15 1299–1309. 10.1111/j.1582-4934.2011.01301.x 21418513 PMC4373330

[B28] Dai X. Yan X. Zeng J. Chen J. Wang Y. Chen J. (2017). Elevating CXCR7 improves angiogenic function of EPCs via Akt/GSK-3β/Fyn-mediated Nrf2 activation in diabetic limb ischemia. *Circ. Res.* 120 e7–e23. 10.1161/CIRCRESAHA.117.310619 28137917 PMC5336396

[B29] Datema R. Rabin L. Hincenbergs M. Moreno M. B. Warren S. Linquist V. (1996). Antiviral efficacy in vivo of the anti-human immunodeficiency virus bicyclam SDZ SID 791 (JM 3100), an inhibitor of infectious cell entry. *Antimicrob. Agents Chemother.* 40 750–754. 8851605 10.1128/aac.40.3.750 PMC163192

[B30] David J. R. (1966). Delayed hypersensitivity in vitro: its mediation by cell-free substances formed by lymphoid cell-antigen interaction. *Proc. Natl. Acad. Sci. U.S.A.* 56 72–77. 10.1073/pnas.56.1.72 5229858 PMC285677

[B31] Davis T. P. Hoyer G. L. Davis P. Burks T. F. (1990). Proenkephalin a-derived peptide E and its fragments alter opioid contractility in the small intestine. *Eur*. *J. Pharmacol.* 191 253–261. 10.1016/0014-2999(90)94157-S 2086244

[B32] De Clercq E. (2005). Potential clinical applications of the CXCR4 antagonist bicyclam AMD3100. *Mini Rev. Med. Chem.* 5 805–824. 10.2174/1389557054867075 16178723

[B33] Décaillot F. M. Kazmi M. A. Lin Y. Ray-Saha S. Sakmar T. P. Sachdev P. (2011). CXCR7/CXCR4 heterodimer constitutively recruits β-arrestin to enhance cell migration. *J. Biol. Chem.* 286 32188–32197. 10.1074/jbc.M111.277038 21730065 PMC3173186

[B34] Dong X. Han S. K. Zylka M. J. Simon M. I. Anderson D. J. (2001). A diverse family of GPCRs expressed in specific subsets of nociceptive sensory neurons. *Cell* 106 619–632. 10.1016/S0092-8674(01)00483-4 11551509

[B35] Dores R. M. McDonald L. K. Steveson T. C. Sei C. A. (1990). The molecular evolution of neuropeptides: prospects for the ’90s. *Brain Behav*. *Evol.* 36 80–99. 10.1159/000115300 2176909

[B36] Dray A. Nunan L. Wire W. (1985). Proenkephalin a fragments exhibit spinal and supraspinal opioid activity in vivo. *J. Pharmacol. Exp. Ther.* 235 670–676. 3001272

[B37] Drury L. J. Ziarek J. J. Gravel S. Veldkamp C. T. Takekoshi T. Hwang S. T. (2011). Monomeric and dimeric CXCL12 inhibit metastasis through distinct CXCR4 interactions and signaling pathways. *Proc*. *Natl. Acad. Sci. U.S.A.* 108 17655–17660. 10.1073/pnas.1101133108 21990345 PMC3203819

[B38] Duda D. G. Kozin S. V. Kirkpatrick N. D. Xu L. Fukumura D. Jain R. K. (2011). CXCL12 (SDF1α)-CXCR4/CXCR7 pathway inhibition: an emerging sensitizer for anticancer therapies? *Clin. Cancer Res.* 17 2074–2080. 10.1158/1078-0432.CCR-10-2636 21349998 PMC3079023

[B39] Dunworth W. P. Fritz-Six K. L. Caron K. M. (2008). Adrenomedullin stabilizes the lymphatic endothelial barrier in vitro and in vivo. *Peptides* 29 2243–2249. 10.1016/j.peptides.2008.09.009 18929609 PMC2639781

[B40] Esencay M. Sarfraz Y. Zagzag D. (2013). CXCR7 is induced by hypoxia and mediates glioma cell migration towards SDF-1α. *BMC Cancer* 13:347. 10.1186/1471-2407-13-347 23865743 PMC3728118

[B41] Fritz-Six K. L. Dunworth W. P. Li M. Caron K. M. (2008). Adrenomedullin signaling is necessary for murine lymphatic vascular development. *J. Clin. Investig.* 118 40–50. 10.1172/JCI33302 18097475 PMC2147672

[B42] Garzon J. Sanchez-Blazquez P. Höllt V. Lee N. M. Loh H. H. (1983). Endogenous opioid peptides: comparative evaluation of their receptor affinities in the mouse brain. *Life Sci.* 33 291–294. 10.1016/0024-3205(83)90500-3 6319879

[B43] Gatti M. Pattarozzi A. Bajetto A. Würth R. Daga A. Fiaschi P. (2013). Inhibition of CXCL12/CXCR4 autocrine/paracrine loop reduces viability of human glioblastoma stem-like cells affecting self-renewal activity. *Toxicology* 314 209–220. 10.1016/j.tox.2013.10.003 24157575

[B44] Geras-Raaka E. Varma A. Clark-Lewis I. Gershengorn M. C. (1998). Kaposi’s sarcoma-associated herpesvirus (KSHV) chemokine vMIP-II and human SDF-1alpha inhibit signaling by KSHV G protein-coupled receptor. *Biochem. Biophys. Res. Commun.* 253 725–727. 10.1006/bbrc.1998.9557 9918794

[B45] Gerrits H. van Ingen Schenau D. S. Bakker N. E. Van Disseldorp A. J. Strik A. Hermens L. S. (2008). Early postnatal lethality and cardiovascular defects in CXCR7-deficient mice. *Genesis* 46 235–245. 10.1002/dvg.20387 18442043

[B46] Goguet-Surmenian E. Richard-Fiardo P. Guillemot E. Benchetrit M. Gomez-Brouchet A. Buzzo P. (2013). CXCR7-mediated progression of osteosarcoma in the lungs. *Br. J. Cancer* 109 1579–1585. 10.1038/bjc.2013.482 24002596 PMC3776992

[B47] Graham G. J. Locati M. Mantovani A. Rot A. Thelen M. (2012). The biochemistry and biology of the atypical chemokine receptors. *Immunol. Lett.* 145 30–38. 10.1016/j.imlet.2012.04.004 22698181

[B48] Gravel S. Malouf C. Boulais P. E. Berchiche Y. A. Oishi S. Fujii N. (2010). The peptidomimetic CXCR4 antagonist TC14012 recruitsβ-Arrestin to CXCR7: roles of receptor domains. *J. Biol. Chem.* 285 37939–37943. 10.1074/jbc.C110.147470 20956518 PMC2992227

[B49] Guan S. Zhou J. (2017). CXCR7 attenuates the TGF-β-induced endothelial-to-mesenchymal transition and pulmonary fibrosis. *Mol. Biosyst.* 13 2116–2124. 10.1039/C7MB00247E 28820530

[B50] Hachet-Haas M. Balabanian K. Rohmer F. Pons F. Franchet C. Lecat S. (2008). Small neutralizing molecules to inhibit actions of the chemokine CXCL12. *J. Biol. Chem.* 283 23189–23199. 10.1074/jbc.M803947200 18556651

[B51] Hao H. Hu S. Chen H. Bu D. Zhu L. Xu C. (2017). Loss of endothelial CXCR7 impairs vascular homeostasis and cardiac remodeling after myocardial infarction: implications for cardiovascular drug discovery. *Circulation* 135 1253–1264. 10.1161/CIRCULATIONAHA.116.023027 28154007

[B52] Hartmann T. N. Grabovsky V. Pasvolsky R. Shulman Z. Buss E. C. Spiegel A. (2008). A crosstalk between intracellular CXCR7 and CXCR4 involved in rapid CXCL12-triggered integrin activation but not in chemokine-triggered motility of human T lymphocytes and CD34^+^ cells. *J. Leukoc. Biol.* 84 1130–1140. 10.1189/jlb.0208088 18653785

[B53] Hattermann K. Held-Feindt J. Lucius R. Müerköster S. S. Penfold M. E. Schall T. J. (2010). The chemokine receptor CXCR7 is highly expressed in human glioma cells and mediates antiapoptotic effects. *Cancer Res.* 70 3299–3308. 10.1158/0008-5472.CAN-09-3642 20388803

[B54] Heesen M. Berman M. A. Charest A. Housman D. Gerard C. Dorf M. E. (1998). Cloning and chromosomal mapping of an orphan chemokine receptor: mouse RDC1. *Immunogenetics* 47 364–370. 10.1007/s002510050371 9510554

[B55] Heinrich E. L. Lee W. Lu J. Lowy A. M. Kim J. (2012). Chemokine CXCL12 activates dual CXCR4 and CXCR7-mediated signaling pathways in pancreatic cancer cells. *J. Transl. Med.* 10:68. 10.1186/1479-5876-10-68 22472349 PMC3342892

[B56] Hernandez L. Magalhaes M. A. Coniglio S. J. Condeelis J. S. Segall J. E. (2011). Opposing roles of CXCR4 and CXCR7 in breast cancer metastasis. *Breast Cancer Res.* 13:R128. 10.1186/bcr3074 22152016 PMC3326570

[B57] Höllt V. Haarmann I. Grimm C. Herz A. Tulunay F. C. Loh H. H. (1982). Pro-enkephalin intermediates in bovine brain and adrenal medulla: characterization of immunoreactive peptides related to BAM-22P and peptide F. *Life Sci.* 31 1883–1886. 10.1016/0024-3205(82)90234-X 7154841

[B58] Hoopes S. L. Willcockson H. H. Caron K. M. (2012). Characteristics of multi-organ lymphangiectasia resulting from temporal deletion of calcitonin receptor-like receptor in adult mice. *PLoS One* 7:e45261. 10.1371/journal.pone.0045261 23028890 PMC3444480

[B59] Ikeda Y. Kumagai H. Skach A. Sato M. Yanagisawa M. (2013). Modulation of circadian glucocorticoid oscillation via adrenal opioid-CXCR7 signaling alters emotional behavior. *Cell* 155 1323–1336. 10.1016/j.cell.2013.10.052 24315101 PMC3934808

[B60] Imamura K. Nishihira J. Suzuki M. Yasuda K. Sasaki S. Kusunoki Y. (1996). Identification and immunohistochemical localization of macrophage migration inhibitory factor in human kidney. *IUBMB Life* 40 1233–1242. 10.1080/15216549600201883 8988336

[B61] Ishii T. Nishihara M. Ma F. Ebihara Y. Tsuji K. Asano S. (1999). Expression of stromal cell-derived factor-1/pre-B cell growth-stimulating factor receptor, CXC chemokine receptor 4, on CD34+ human bone marrow cells is a phenotypic alteration for committed lymphoid progenitors. *J. Immunol.* 163 3612–3620. 10490954

[B62] Juarez J. Bendall L. Bradstock K. (2004). Chemokines and their receptors as therapeutic targets: the role of the SDF-1/CXCR4 axis. *Curr. Pharm. Des.* 10 1245–1259. 10.2174/1381612043452640 15078139

[B63] Kalatskaya I. Berchiche Y. A. Gravel S. Limberg B. J. Rosenbaum J. S. Heveker N. (2009). AMD3100 is a CXCR7 ligand with allosteric agonist properties. *Mol. Pharmacol.* 75 1240–1247. 10.1124/mol.108.053389.luciferase 19255243

[B64] Kapas S. Clark A. J. (1995). Identification of an orphan receptor gene as a type 1 calcitonin gene-related peptide receptor. *Biochem. Biophys. Res. Commun.* 217 832–838. 10.1006/bbrc.1995.2847 8554605

[B65] Karin N. (2010). The multiple faces of CXCL12 (SDF-1alpha) in the regulation of immunity during health and disease. *J. Leukoc. Biol.* 88 463–473. 10.1189/jlb.0909602 20501749

[B66] Karpinich N. O. Hoopes S. L. Kechele D. O. Lenhart P. M. Caron K. M. (2011). Adrenomedullin function in vascular endothelial cells: insights from genetic mouse models. *Curr. Hypertens. Rev.* 7 228–239. 10.2174/157340211799304761 22582036 PMC3349984

[B67] Khachaturian H. Lewis M. E. (1983). Telencephalic enkephalinergic systems in the rat brain. *J. Neurosci.* 3 844–855. 10.1523/JNEUROSCI.03-04-00844.1983 6834107 PMC6564446

[B68] Kitamura K. Kangawa K. Kawamoto M. Ichiki Y. Nakamura S. Matsuo H. (1993). Adrenomedullin: a novel hypotensive peptide isolated from human pheochromocytoma. *Biochem. Biophys. Res. Commun.* 192 553–560. 10.1006/bbrc.1993.1451 8387282

[B69] Kledal T. N. Rosenkilde M. M. Coulin F. Simmons G. Johnsen A. H. Alouani S. (1997). A broad-spectrum chemokine antagonist encoded by kaposi’s sarcoma-associated herpesvirus. *Science* 277 1656–1659. 10.1126/science.277.5332.1656 9287217

[B70] Klein K. R. Karpinich N. O. Espenschied S. T. Willcockson H. H. Dunworth W. P. Hoopes S. L. (2014). Decoy receptor CXCR7 modulates adrenomedullin-mediated cardiac and lymphatic vascular development. *Dev. Cell* 30 528–540. 10.1016/j.devcel.2014.07.012 25203207 PMC4166507

[B71] Koshiba T. Hosotani R. Miyamoto Y. Ida J. Tsuji S. Nakajima S. (2000). Expression of stromal cell-derived factor 1 and CXCR4 ligand receptor system in pancreatic cancer: a possible role for tumor progression. *Clin. Cancer Res.* 6 3530–3535. 10999740

[B72] Lang T. Foote A. Lee J. P. Morand E. F. Harris J. (2015). MIF: implications in the pathoetiology of systemic lupus erythematosus. *Front. Immunol.* 6:577. 10.3389/fimmu.2015.00577 26617609 PMC4641160

[B73] Lasagni L. Francalanci M. Annunziato F. Lazzeri E. Giannini S. Cosmi L. (2003). An alternatively spliced variant of CXCR3 mediates the inhibition of endothelial cell growth induced by IP-10, Mig, and I-TAC, and acts as functional receptor for platelet factor 4. *J. Exp. Med.* 197 1537–1549. 10.1084/jem.20021897 12782716 PMC2193908

[B74] Lataillade J. J. Domenech J. Le Bousse-Kerdilès M. C. (2004). Stromal cell-derived factor-1 (SDF-1)/CXCR4 couple plays multiple roles on haematopoietic progenitors at the border between the old cytokine and new chemokine worlds: survival, cell cycling and trafficking. *Eur. Cytok. Netw.* 15 177–188. 15542441

[B75] Lembo P. M. Grazzini E. Groblewski T. O’Donnell D. Roy M. O. Zhang J. (2002). Proenkephalin a gene products activate a new family of sensory neuron–specific GPCRs. *Nat. Neurosci.* 5 201–209. 10.1038/nn815 11850634

[B76] Leng L. Metz C. N. Fang Y. Xu J. Donnelly S. Baugh J. (2003). MIF signal transduction initiated by binding to CD74. *J. Exp. Med.* 197 1467–1476. 10.1084/jem.20030286 12782713 PMC2193907

[B77] Levoye A. Balabanian K. Baleux F. Bachelerie F. Lagane B. (2009). CXCR7 heterodimerizes with CXCR4 and regulates CXCL12-mediated G protein signaling. *Blood* 113 6085–6093. 10.1182/blood-2008-12-196618 19380869

[B78] Li M. Ransohoff R. M. (2009). The roles of chemokine CXCL12 in embryonic and brain tumor angiogenesis. *Semin. Cancer Biol.* 19 111–115. 10.1016/j.semcancer.2008.11.001 19038344

[B79] Liang Z. Wu H. Reddy S. Zhu A. Wang S. Blevins D. (2007). Blockade of invasion and metastasis of breast cancer cells via targeting CXCR4 with an artificial microRNA. *Biochem. Biophys. Res. Commun.* 363 542–546. 10.1016/j.bbrc.2007.09.007 17889832

[B80] Liberman J. Sartelet H. Flahaut M. Mühlethaler-Mottet A. Coulon A. Nyalendo C. (2012). Involvement of the CXCR7/CXCR4/CXCL12 axis in the malignant progression of human neuroblastoma. *PLoS One* 7:e43665. 10.1371/journal.pone.0043665 22916293 PMC3423387

[B81] Libert F. Parmentier M. Lefort A. Dinsart C. Van Sande J. Maenhaut C. (1989). Selective amplification and cloning of four new members of the G protein-coupled receptor family. *Science* 244 569–572. 10.1126/science.2541503 2541503

[B82] Libert F. Parmentier M. Lefort A. Dumont J. E. Vassart G. (1990). Complete nucleotide sequence of a putative G protein coupled receptor: RDC7. *Nucleic Acids Res.* 18:1915. 10.1093/nar/18.7.1915 2159629 PMC330638

[B83] Lin C.-H. Shih C.-H. Tseng C.-C. Yu C.-C. Tsai Y.-J. Bien M.-Y. (2014). CXCL12 induces connective tissue growth factor expression in human lung fibroblasts through the Rac1/ERK, JNK, and AP-1 pathways. *PLoS One* 9:e104746. 10.1371/journal.pone.0104746 25121739 PMC4133236

[B84] Liu H. Xue W. Ge G. Luo X. Li Y. Xiang H. (2010). Hypoxic preconditioning advances CXCR4 and CXCR7 expression by activating HIF-1α in MSCs. *Biochem. Biophys. Res. Commun.* 401 509–515. 10.1016/j.bbrc.2010.09.076 20869949

[B85] Liu L. Zhao X. Zhu X. Zhong Z. Xu R. Wang Z. (2013). Decreased expression of miR-430 promotes the development of bladder cancer via the upregulation of CXCR7. *Mol. Med. Rep.* 8 140–146. 10.3892/mmr.2013.1477 23677384

[B86] Liu S. C. Alomran R. Chernikova S. B. Lartey F. Stafford J. Jang T. (2014). Blockade of SDF-1 after irradiation inhibits tumor recurrences of autochthonous brain tumors in rats. *Neuro Oncol.* 16 21–28. 10.1093/neuonc/not149 24335554 PMC3870826

[B87] Liu T. J. Guo J. L. Xu X. (2017). CXC chemokine-7 inhibits growth and migration of oral tongue squamous cell carcinoma cells, mediated by the epithelial-mesenchymal transition signaling pathway. *Mol. Med. Rep.* 16 6896–6903. 10.3892/mmr.2017.7441 28901471

[B88] Lue H. Thiele M. Franz J. Dahl E. Speckgens S. Leng L. (2007). Macrophage migration inhibitory factor (MIF) promotes cell survival by activation of the Akt pathway and role for CSN5/JAB1 in the control of autocrine MIF activity. *Oncogene* 26 5046–5059. 10.1038/sj.onc.1210318 17310986

[B89] Luker K. E. Lewin S. A. Mihalko L. A. Schmidt B. T. Winkler J. S. Coggins N. L. (2012). Scavenging of CXCL12 by CXCR7 promotes tumor growth and metastasis of CXCR4-positive breast cancer cells. *Oncogene* 31 4750–4758. 10.1038/onc.2011.633 22266857 PMC3337948

[B90] Lüttichau H. R. Johnsen A. H. Jurlander J. Rosenkilde M. M. Schwartz T. W. (2007). Kaposi sarcoma-associated herpes virus targets the lymphotactin receptor with both a broad spectrum antagonist vCCL2 and a highly selective and potent agonist vCCL3. *J. Biol. Chem.* 282 17794–17805. 10.1074/jbc.M702001200 17403668

[B91] Ma W. Liu Y. Ellison N. Shen J. (2013). Induction of C-X-C chemokine receptor type 7 (CXCR7) switches stromal cell-derived factor-1 (SDF-1) signaling and phagocytic activity in macrophages linked to atherosclerosis. *J. Biol. Chem.* 288 15481–15494. 10.1074/jbc.M112.445510 23599431 PMC3668710

[B92] Ma W. Liu Y. Wang C. Zhang L. Crocker L. Shen J. (2014). Atorvastatin inhibits CXCR7 induction to reduce macrophage migration. *Biochem*. *Pharmacol.* 89 99–108. 10.1016/j.bcp.2014.02.014 24582769

[B93] McLatchie L. M. Fraser N. J. Main M. J. Wise A. Brown J. Thompson N. (1998). RAMPs regulate the transport and ligand specificity of the calcitonin-receptor-like receptor. *Nature* 393 333–339. 10.1038/30666 9620797

[B94] Melo R. C. C. Ferro K. P. V. Duarte A. D. S. S. Olalla Saad S. T. (2018). CXCR7 participates in CXCL12-mediated migration and homing of leukemic and normal hematopoietic cells. *Stem Cell Res. Ther.* 9:34. 10.1186/s13287-017-0765-1 29433559 PMC5810108

[B95] Miao Z. Luker K. E. Summers B. C. Berahovich R. Bhojani M. S. Rehemtulla A. (2007). CXCR7 (RDC1) promotes breast and lung tumor growth in vivo and is expressed on tumor-associated vasculature. *Proc*. *Natl. Acad. Sci. U.S.A.* 104 15735–15740. 10.1073/pnas.0610444104 17898181 PMC1994579

[B96] Mizuno K. Minamino N. Kangawa K. Matsuo H. (1980). A new endogenous opioid peptide from bovine adrenal medulla: isolation and amino acid sequence of a dodecapeptide (BAM-12P). *Biochem. Biophys. Res. Commun.* 95 1482–1488. 10.1016/S0006-291X(80)80064-7 7417331

[B97] Montpas N. Cabana J. St-Onge G. Gravel S. Morin G. Kuroyanagi T. (2015). Mode of binding of the cyclic agonist peptide TC14012 to CXCR7: identification of receptor and compound determinants. *Biochemistry* 54 1505–1515. 10.1021/bi501526s 25669416

[B98] Moore P. S. Boshoff C. Weiss R. A. Chang Y. (1996). Molecular mimicry of human cytokine and cytokine response pathway genes by KSHV. *Science* 274 1739–1744. 10.1126/science.274.5293.1739 8939871

[B99] Morand E. F. Leech M. Bernhagen J. (2006). MIF: a new cytokine link between rheumatoid arthritis and atherosclerosis. *Nat. Rev. Drug Discov.* 5 399–410. 10.1038/nrd2029 16628200

[B100] Nagasawa T. Nakajima T. Tachibana K. Iizasa H. Bleul C. C. Yoshie O. (1996). Molecular cloning and characterization of a murine Pre-B-cell growth-stimulating factor/stromal cell-derived factor 1 receptor, a murine homolog of the human immunodeficiency virus 1 entry coreceptor fusin. *Proc. Natl. Acad. Sci. U.S.A.* 93 14726–14729. 10.1073/pnas.93.25.14726 8962122 PMC26203

[B101] Nanki T. Lipsky P. E. (2000). Cutting edge: stromal cell-derived factor-1 is a costimulator for CD4+ T cell activation. *J. Immunol.* 164 5010–5014. 10.4049/jimmunol.164.10.5010 10799853

[B102] Naumann U. Cameroni E. Pruenster M. Mahabaleshwar H. Raz E. Zerwes H. G. (2010). CXCR7 functions as a scavenger for CXCL12 and CXCL11. *PLoS One* 5:e9175. 10.1371/journal.pone.0009175 20161793 PMC2820091

[B103] Nicholas J. Ruvolo V. Zong J. Ciufo D. Guo H. G. Reitz M. S. (1997). A Single 13-kilobase divergent locus in the kaposi sarcoma-associated herpesvirus (human herpesvirus 8) genome contains nine open reading frames that are homologous to or related to cellular proteins. *J. Virol.* 71 1963–1974. 9032328 10.1128/jvi.71.3.1963-1974.1997 PMC191280

[B104] Nishihira J. Koyama Y. Mizue Y. (1998). Identification of macrophage migration inhibitory factor (Mif) in human vascular enothelial cells and its induction by lipopolysaccharide. *Cytokine* 10 199–205. 9576065 10.1006/cyto.1997.0276

[B105] Odemis V. Lipfert J. Kraft R. Hajek P. Abraham G. Hattermann K. (2012). The presumed atypical chemokine receptor CXCR7 signals through Gi/o proteins in primary rodent astrocytes and human glioma cells. *Glia* 60 372–381. 10.1002/glia.22271 22083878

[B106] Pan F. Ma S. Cao W. Liu H. Chen F. Chen X. (2013). SDF-1alpha upregulation of MMP-2 is mediated by p38 MAPK signaling in pancreatic cancer cell lines. *Mol. Biol. Rep.* 40 4139–4146. 10.1007/s11033-012-2225-4 23712777

[B107] Pittius C. W. Seizinger B. R. Pasi A. Mehraein P. Herz A. (1984). Distribution and characterization of opioid peptides derived from proenkephalin a in human and rat central nervous system. *Brain Res.* 304 127–136. 6744032 10.1016/0006-8993(84)90868-0

[B108] Proost P. Mortier A. Loos T. Vandercappellen J. Gouwy M. Ronsse I. (2007). Proteolytic processing of CXCL11 by CD13/aminopeptidase N impairs CXCR3 and CXCR7 binding and signaling and reduces lymphocyte and endothelial cell migration. *Blood* 110 37–44. 17363734 10.1182/blood-2006-10-049072

[B109] Qin L. Kufareva I. Holden L. G. Wang C. Zheng Y. Zhao C. (2015). Structural biology. Crystal structure of the chemokine receptor CXCR4 in complex with a viral chemokine. *Science* 347 1117–1122. 10.1126/science.1261064 25612609 PMC4362693

[B110] Quirion R. Weiss A. S. (1983). Peptide E and other proenkephalin-derived peptides are potent kappa opiate receptor agonists. *Peptides* 4 445–449. 6316294 10.1016/0196-9781(83)90047-5

[B111] Rajagopal S. Kim J. Ahn S. Craig S. Lam C. M. Gerard N. P. (2010). Beta-arrestin- but not G protein-mediated signaling by the ‘decoy’ receptor CXCR7. *Proc*. *Natl. Acad. Sci. U.S.A.* 107 628–632. 10.1073/pnas.0912852107 20018651 PMC2818968

[B112] Ray P. Lewin S. A. Mihalko L. A. Lesher-Perez S. C. Takayama S. Luker K. E. (2012). Secreted CXCL12 (SDF-1) forms dimers under physiological conditions. *Biochem. J.* 442 433–442. 10.1042/BJ20111341 22142194 PMC4419379

[B113] Rollins B. J. (1997). Chemokines. *Blood* 90 909–928. 10.1111/j.1365-2036.2010.04439.x 9242519

[B114] Romagnani P. Lasagni L. Annunziato F. Serio M. Romagnani S. (2004). CXC chemokines: the regulatory link between inflammation and angiogenesis. *Trends Immunol.* 25 201–209. 10.1016/j.it.2004.02.006 15039047

[B115] Rossi A. G. Haslett C. Hirani N. Greening A. P. Rahman I. Metz C. N. (1998). Human circulating eosinophils secrete macrophage migration inhibitory factor (MIF): potential role in asthma. *J. Clin. Investig.* 101 2869–2874. 10.1172/JCI1524 9637721 PMC508878

[B116] Sánchez-Blázquez P. Garzón J. (1985). Opioid activity of pro-enkephalin-derived peptides in mouse vas deferens and guinea pig ileum. *Neurosci. Lett.* 61 267–271. 3001596 10.1016/0304-3940(85)90475-6

[B117] Schutyser E. Su Y. Yu Y. Gouwy M. Zaja-Milatovic S. Van Damme J. (2007). Hypoxia enhances CXCR4 expression in human microvascular endothelial cells and human melanoma cells. *Eur*. *Cytokine Netw.* 18 59–70. 10.1684/ecn.2007.0087 17594938 PMC2665278

[B118] Schwartz V. Lue H. Kraemer S. Korbiel J. Krohn R. Ohl K. (2009). A functional heteromeric MIF receptor formed by CD74 and CXCR4. *FEBS Lett.* 583 2749–2757. 10.1016/j.febslet.2009.07.058 19665027 PMC2911026

[B119] Sekiguchi H. Kuroyanagi T. Rhainds D. Kobayashi K. Kobayashi Y. Ohno H. (2018). Structure–activity relationship study of cyclic pentapeptide ligands for atypical chemokine receptor 3 (ACKR3). *J. Med. Chem.* 61 3745–3751. 10.1021/acs.jmedchem.8b00336 29608300

[B120] Shi X. Leng L. Wang T. Wang W. Du X. Li J. (2006). CD44 is the signaling component of the macrophage migration inhibitory factor-CD74 receptor complex. *Immunity* 25 595–606. 10.1016/j.immuni.2006.08.020 17045821 PMC3707630

[B121] Sierro F. Biben C. Martínez-Muñoz L. Mellado M. Ransohoff R. M. Li M. (2007). Disrupted cardiac development but normal hematopoiesis in mice deficient in the second CXCL12/SDF-1 receptor, CXCR7. *Proc. Natl. Acad. Sci. U.S.A.* 104 14759–14764. 10.1073/pnas.0702229104 17804806 PMC1976222

[B122] Singh R. K. Lokeshwar B. L. (2011). The IL-8-regulated chemokine receptor CXCR7 stimulates EGFR signaling to promote prostate cancer growth. *Cancer Res.* 71 3268–3277. 10.1158/0008-5472.CAN-10-2769 21398406 PMC3085571

[B123] Staton A. A. Knaut H. Giraldez A. J. (2011). miRNA regulation of Sdf1 chemokine signaling provides genetic robustness to germ cell migration. *Nat. Genet.* 43 204–211. 10.1038/ng.758 21258340 PMC3071589

[B124] Strieter R. M. Burdick M. D. Gomperts B. N. Belperio J. A. Keane M. P. (2005). CXC chemokines in angiogenesis. *Cytokine Growth Factor Rev.* 16 593–609. 10.1016/j.cytogfr.2005.04.007 16046180

[B125] Szpakowska M. Chevigné A. (2015). vCCL2/vMIP-II, the viral master KEYmokine. *J. Leukoc. Biol.* 99 893–900. 10.1189/jlb.2MR0815-383R 26701133

[B126] Szpakowska M. Dupuis N. Baragli A. Counson M. Hanson J. Piette J. (2016). Human herpesvirus 8-encoded chemokine vCCL2/vMIP-II is an agonist of the atypical chemokine receptor ACKR3/CXCR7. *Biochem. Pharmacol.* 114 14–21. 10.1016/j.bcp.2016.05.012 27238288

[B127] Tamamura H. Omagari A. Hiramatsu K. Gotoh K. Kanamoto T. Xu Y. (2001). Development of specific CXCR4 inhibitors possessing high selectivity indexes as well as complete stability in serum based on an anti-HIV peptide T140. *Bioorg. Med. Chem. Lett.* 11 1897–1902. 11459656 10.1016/s0960-894x(01)00323-7

[B128] Tamamura H. Xu Y. Hattori T. Zhang X. Arakaki R. Kanbara K. (1998). A low-molecular-weight inhibitor against the chemokine receptor CXCR4: a strong anti-HIV peptide T140. *Biochem. Biophys. Res. Commun.* 253 877–882. 10.1006/bbrc.1998.9871 9918823

[B129] Tarnowski M. Grymula K. Liu R. Tarnowska J. Drukala J. Ratajczak J. (2010a). Macrophage migration inhibitory factor is secreted by rhabdomyosarcoma cells, modulates tumor metastasis by binding to CXCR4 and CXCR7 receptors and inhibits recruitment of cancer-associated fibroblasts. *Mol. Cancer Res.* 8 1328–1343. 10.1158/1541-7786.MCR-10-0288 20861157 PMC2974061

[B130] Tarnowski M. Liu R. Wysoczynski M. Ratajczak J. Kucia M. Ratajczak M. Z. (2010b). CXCR7: a new SDF-1-binding receptor in contrast to normal CD34+ progenitors is functional and is expressed at higher level in human malignant hematopoietic cells. *Eur. J. Haematol.* 85 472–483. 10.1111/j.1600-0609.2010.01531.x 20887389

[B131] Teng F. Tian W. Y. Wang Y. M. Zhang Y. F. Guo F. Zhao J. (2016). Cancer-associated fibroblasts promote the progression of endometrial cancer via the SDF-1/CXCR4 axis. *J. Hematol. Oncol.* 9:8. 10.1186/s13045-015-0231-4 26851944 PMC4744391

[B132] To L. B. Levesque J. P. Herbert K. E. (2011). How I treat patients who mobilize hematopoietic stem cells poorly. *Blood* 118 4530–4540. 10.1182/blood-2011-06-318220 21832280

[B133] Trent J. O. Wang Z. X. Murray J. L. Shao W. Tamamura H. Fujii N. (2003). Lipid bilayer simulations of CXCR4 with inverse agonists and weak partial agonists. *J. Biol. Chem.* 278 47136–47144. 10.1074/jbc.M307850200 12958314

[B134] Uto-Konomi A. McKibben B. Wirtz J. Sato Y. Takano A. Nanki T. (2013). CXCR7 agonists inhibit the function of CXCL12 by down-regulation of CXCR4. *Biochem. Biophys. Res. Commun.* 431 772–776. 10.1016/j.bbrc.2013.01.032 23333329

[B135] Van Rechem C. Rood B. R. Touka M. Pinte S. Jenal M. Guérardel C. (2009). Scavenger chemokine (CXC Motif) receptor 7 (CXCR7) is a direct target gene of HIC1 (hypermethylated in cancer 1). *J. Biol. Chem.* 284 20927–20935. 10.1074/jbc.M109.022350 19525223 PMC2742858

[B136] Vandercappellen J. Van Damme J. Struyf S. (2008). The role of CXC chemokines and their receptors in cancer. *Cancer Lett.* 267 226–244. 10.1016/j.canlet.2008.04.050 18579287

[B137] Vischer H. F. Siderius M. Leurs R. Smit M. J. (2014). Herpesvirus-encoded GPCRs: neglected players in inflammatory and proliferative diseases? *Nat. Rev. Drug Discov.* 13 123–139. 10.1038/nrd4189 24445563

[B138] Wang H. Beaty N. Chen S. Qi C. F. Masiuk M. Shin D. M. (2012). The CXCR7 chemokine receptor promots B-cell retention in the splenic marginal zone and serves as a sink for CXCL12. *Blood* 119 465–468. 10.1182/blood-2011-03-343608 22110250 PMC3257011

[B139] Wang J. Shiozawa Y. Wang J. Wang Y. Jung Y. Pienta K. J. (2008). The role of CXCR7/RDC1 as a chemokine receptor for CXCL12/SDF-1 in prostate cancer. *J. Biol. Chem.* 283 4283–4294. 18057003 10.1074/jbc.M707465200

[B140] Wang L. Zhang X. Jia L. T. Hu S. J. Zhao J. Yang J. D. (2014). C-Myc-mediated epigenetic silencing of MicroRNA-101 contributes to dysregulation of multiple pathways in hepatocellular carcinoma. *Hepatology* 59 1850–1863. 10.1002/hep.26720 24002871

[B141] Wanshu M. Liu Y. Ellison N. Shen J. (2013). Induction of C-X-C chemokine receptor type 7 (CXCR7) switches stromal cell-derived factor-1 (SDF-1) signaling and phagocytic activity in macrophages linked to atherosclerosis. *J. Biol. Chem.* 288 15481–15494. 10.1074/jbc.M112.445510 23599431 PMC3668710

[B142] Wetzel-Strong S. E. Li M. Klein K. R. Nishikimi T. Caron K. M. (2014). Epicardial-derived adrenomedullin drives cardiac hyperplasia during embryogenesis. *Dev. Dyn.* 243 243–256. 10.1002/dvdy.24065 24123312 PMC4009724

[B143] Wijtmans M. Maussang D. Sirci F. Scholten D. J. Canals M. Mujić-Delić A. (2012). Synthesis, modeling and functional activity of substituted styrene-amides as small-molecule CXCR7 agonists. *Eur. J. Med. Chem.* 51 184–192. 10.1016/j.ejmech.2012.02.041 22424612

[B144] Wu B. Chien E. Y. T. Mol C. D. Fenalti G. Liu W. Katritch V. (2010). Structures of the CXCR4 chemokine GPCR with small-molecule and cyclic peptide antagonists. *Science* 330 1066–1071. 10.1126/science.1194396 20929726 PMC3074590

[B145] Wu Q. Dhir R. Wells A. (2012). Altered CXCR3 isoform expression regulates prostate cancer cell migration and invasion. *Mol. Cancer* 11:3. 10.1186/1476-4598-11-3 22236567 PMC3320557

[B146] Wu W. Qian L. Chen X. Ding B. (2015). Prognostic significance of CXCL12, CXCR4, and CXCR7 in patients with breast cancer. *Int. J. Clin. Exp. Pathol.* 8:13217. PMC4680466 26722521

[B147] Wu Y.-C. Tang S.-J. Sun G.-H. Sun K.-H. (2015). CXCR7 mediates TGFβ1-promoted EMT and tumor-initiating features in lung cancer. *Oncogene* 325 1–10. 10.1038/onc.2015.274 26212008

[B148] Xiao W. Dong X. Zhao H. Han S. Nie R. Zhang X. (2016). MIF signal transduction initiated by binding to CD74. *J. Exp. Med.* 197 1467–1476. 12782713 10.1084/jem.20030286 PMC2193907

[B149] Yamada K. Maishi N. Akiyama K. TowfikAlam K. Ohga N. Kawamoto T. (2015). CXCL12-CXCR7 axis is important for tumor endothelial cell angiogenic property. *Int. J. Cancer* 137 2825–2836. 10.1002/ijc.29655 26100110

[B150] Yan X. Cai S. Xiong X. Sun W. Dai X. Chen S. (2012). Chemokine receptor CXCR7 mediates human endothelial progenitor cells survival, angiogenesis, but not proliferation. *J. Cell. Biochem.* 113 1437–1446. 10.1002/jcb.24015 22173725

[B151] Yoshikawa Y. Oishi S. Kubo T. Tanahara N. Fujii N. Furuya T. (2013). Optimized method of G-protein-coupled receptor homology modeling: its application to the discovery of novel CXCR7 ligands. *J. Med. Chem.* 56 4236–4251. 10.1021/jm400307y 23656360

[B152] Yu S. Crawford D. Tsuchihashi T. Behrens T. W. Srivastava D. (2011). The chemokine receptor CXCR7 functions to regulate cardiac valve remodeling. *Dev. Dyn.* 240 384–393. 10.1002/dvdy.22549 21246655 PMC3079332

[B153] Zabel B. A. Lewén S. Berahovich R. D. Jaén J. C. Schall T. J. (2011). The novel chemokine receptor CXCR7 regulates trans-endothelial migration of cancer cells. *Mol. Cancer* 10:73. 10.1186/1476-4598-10-73 21672222 PMC3123309

[B154] Zabel B. A. Wang Y. Lewén S. Berahovich R. D. Penfold M. E. Zhang P. (2009). Elucidation of CXCR7-mediated signaling events and inhibition of CXCR4-mediated tumor cell transendothelial migration by CXCR7 ligands. *J. Immunol.* 183 3204–3211. 10.4049/jimmunol.0900269 19641136

[B155] Zhao Y. Tan Y. Xi S. Li Y. Li C. Cui J. (2013). A novel mechanism by which Sdf-1b protects cardiac cells from palmitate-induced endoplasmic reticulum stress and apoptosis via CXCR7 and AMPK/p38 MAPK-mediated interleukin-6 generation. *Diabetes Metab. Res. Rev.* 62 2545–2558. 10.2337/db12-1233 23423573 PMC3712029

[B156] Zheng J. Weng J. Sun X. Hao M. Ding T. Xiong D. (2013). HIC1 modulates prostate cancer progression by epigenetic modification. *Clin. Cancer Res.* 19 1400–1410. 10.1158/1078-0432.CCR-12-2888 23340301

[B157] Zheng K. Li H. Y. Su X. L. Wang X. Y. Tian T. Li F. (2010). Chemokine receptor CXCR7 regulates the invasion, angiogenesis and tumor growth of human hepatocellular carcinoma cells. *J. Exp. Clin. Cancer Res.* 29:31. 10.1186/1756-9966-29-31 20380740 PMC2859378

[B158] Zou Y. R. Kottmann A. H. Kuroda M. Taniuchi I. Littman D. R. (1998). Function of the chemokine receptor CXCR4 in haematopoiesis and in cerebellar development. *Nature* 393 595–599. 9634238 10.1038/31269

[B159] Zwiech R. (2015). Macrophage migration inhibitory factor urinary excretion revisited–MIF a potent predictor of the immunosuppressive treatment outcomes in patients with proliferative primary glomerulonephritis. *BMC Immunol.* 16:47. 10.1186/s12865-015-0112-1 26272322 PMC4536780

